# THERAPEUTIC BLOWING TOYS: DOES THE OVERLAP OF VENTILATORY STIMULI ALTER THE RESPIRATORY MECHANICS OF HEALTHY SCHOOLCHILDREN?

**DOI:** 10.1590/1984-0462/2020/38/2018259

**Published:** 2020-03-09

**Authors:** Camila Isabel Santos Schivinski, Bruna Cardoso Manna, Fabíula Joanita da Mata Belém, Tayná Castilho

**Affiliations:** a Universidade do Estado de Santa Catarina, Florianópolis, SC, Brazil.

**Keywords:** Adolescent, Child, Physical therapy specialty, Play and playthings, Respiratory mechanics, Adolescente, Criança, Fisioterapia, Jogos e brinquedos, Mecânica respiratória

## Abstract

**Objective:**

To verify whether the overlapping of ventilatory stimuli, resulting from playing with blowing toys, changes the respiratory mechanics of healthy schoolchildren.

**Methods:**

Cross-sectional study with healthy schoolchildren aged seven to 14 years old from Florianópolis, Santa Catarina, Southern Brazil. Spirometric data were obtained, a health questionnaire and the *International Study of Asthma and Allergies in Childhood* (ISAAC) questionnaire were also applied. The procedure consisted of playing with the following blow toys in a random order: soap bubbles, party whistles and balloon. Before and after the intervention, the assessment of respiratory mechanics was carried out by impulse oscillometry - IOS (Erich Jaeger, Germany^®^). The ANOVA for repeated measures test was applied.

**Results:**

71 students of both genders with mean age of 9.7±2.1 years participated in the study. Results showed a progressive decrease of impedance (Z5), total airway resistance (R5) and resonance frequency (Fres) when the moment before the use of the first toy was compared with the moment after the third toy (Z5/p=0.048; R5/p=0.049; Fres/p=0.004). Fres also differed between the moment before the first and the second toy (p=0.048). After the use of each of the three blowing toys, the oscillometric parameters did not differ.

**Conclusions:**

The difference in oscillometric parameters of R5 before the use of each toy indicates that the overlap of ventilatory stimuli produced by them provided a reduction in the R5.

## RESUMO

**Objetivo:**

Verificar se a sobreposição de estímulos ventilatórios decorrentes da execução de brinquedos de sopro altera a mecânica respiratória de escolares saudáveis.

**Métodos:**

Estudo transversal com escolares saudáveis de sete a 14 anos de idade, provenientes de Florianópolis, Santa Catarina, Brasil. Foram obtidos dados espirométricos e realizada aplicação de um recordatório de saúde e do questionário *International Study of Asthma and Allergies in Childhood* (ISAAC). A coleta de dados consistiu na aplicação dos brinquedos bola de sabão, língua de sogra e balão de forma aleatória. Antes e após a intervenção foi realizada a avaliação da mecânica respiratória por meio da oscilometria de impulso - IOS (Erich Jaeger, Germany^®^). Aplicou-se o teste de ANOVA para medidas repetidas.

**Resultados:**

Participaram do estudo 71 escolares de ambos os sexos, com média de idade de 9,7±2,1 anos. Houve redução progressiva na impedância respiratória a 5 hertz (Z5), na resistência total das vias aéreas (resistência a 5 hertz - R5) e na frequência de ressonância (Fres) ao comparar o momento antes do uso do primeiro e do terceiro brinquedo (Z5/p=0,048; R5/p=0,049; Fres/p=0,004). Fres também diferiu no momento antes do primeiro e do segundo brinquedo (p=0,048). Após o uso de cada um dos três brinquedos, os parâmetros oscilométricos não diferiram.

**Conclusões:**

Observando a diferença nos parâmetros oscilométricos da R5 antes do uso de cada um dos brinquedos, notou-se que a sobreposição de estímulos ventilatórios produzidos por eles proporcionou uma redução na R5.

## INTRODUCTION

Respiratory physiotherapy aims to mobilize airway secretions, make pulmonary ventilation adequate, maintain pulmonary function, and prevent respiratory complications. However, each patient has their specificities and the physical therapist should bear this in mind.^1^ In pediatrics, patients have unique interests and needs and, by evaluating them, the physical therapist must recognize limitations, difficulties and clinical conditions of each individual. Thus, therapeutic strategies should be created to make pediatric care more enjoyable, according to the interests and age of each patient.^2^ ^,^ ^3^

The use of toys by health professionals is one of the means to help the child assimilate what is requested, serving as an instrument of communication and guidance, besides making the therapy more enjoyable. Including these in therapy improves the bond with the professional, promotes fun, relaxation and faster recovery, and favors better adherence to treatment.^4^ ^,^ ^5^

In respiratory physiotherapy, the use of blow toys is frequent because they are facilitators to achieve the treatment goals,^6^ stimulating and optimizing different breathing patterns.^7^ Party whistlers, for example, when inserted in clinical practice, take children to a festive moment and enables them to effectively perform inspiration and exhalation in the simple act of blowing the toy.^8^

During therapy, the physical therapist uses different toys, sometimes randomly, sometimes according to children’s preferences or respecting the therapy’s goal. Despite being part of the routine physical therapy care, the act of “playing” and the use of blow toys have not been planned, since there are no studies on the effectiveness of such resources in bringing benefits for the respiratory system, the sum of their effects. or the importance of order of use regarding possible repercussions on the respiratory mechanics of children.

One instrument that allows the evaluation of respiratory mechanics and can be used to verify the effects of blow toys on the airways is the impulse oscillometry system (IOS), which has been shown to be easy applicable in children because it does not require forced breathing maneuvers.^9^ This system generates oscillatory pressures of different frequencies (5-20 Hz) that are transmitted to the lung tissue, and also measures airway resistance (R) and reactance (X). Commonly analyzed parameters are: R, X, airway impedance (Z), resonance frequency (Fres), and reactance area (AX).^10^ To date, no similar work has been conducted on IOS to analyze the repercussion of blow toys on children’s airways, being patients healthy or presenting respiratory impairment, despite the frequent use of this therapeutic strategy in pediatrics.

In this context, the objective of this paper was to investigate whether the overlap of ventilatory stimuli with toys, regardless of type and order of execution, has an effect on the respiratory mechanics of healthy students.

## METHOD

This is a cross-sectional study with a quasi-experimental before-after branch.^11^ In this convenience sample, healthy students of both sexes, aged 7 to 14 years, were selected to participate. After invitation and prior contact by researchers with public and private schools in Greater Florianópolis, Santa Catarina, Brazil, the students’ caregivers/guardians interested in participating, scheduled the date for evaluation, which took place at the physical therapy school-clinic facility of Universidade do Estado de Santa Catarina (Udesc). The study was approved by the Research Ethics Committee (CEP) of Udesc (CAAE: 52891215.7.0000.0118), and registered on the Brazilian Clinical Trial Registry (ReBEC) website, under number RBR-96MZ5C.

All participants interested in the research underwent evaluation for sample selection, but only healthy, oriented and collaborative children and adolescents born at term without invasive mechanical pulmonary ventilation in the neonatal period, without any cardiorespiratory or rheumatic, musculoskeletal, neurological diseases, visual or hearing deficits were included. This information was obtained by means of a health form (prepared by the researchers) sent by the school to parents and/or guardians, along with the International Study of Asthma and Allergies in Childhood (ISAAC) questionnaire.^12^ ^,^ ^13^ Module 1 of ISAAC I for asthma identification was applied, and the cutoff value was a score higher than five for children aged six to nine years, and higher than six for children aged 10 to 14 years for identification of the disease. Spirometry was also required (EasyOne^®^ Medizintechnik AG, Zurich) and performed according to the American Thoracic Society guidelines,^14^ with a minimum of three and maximum of eight maneuvers, forced expiratory volume values ​​in one second (FEV_1_) and forced vital capacity (FVC) above 80% of the predicted,^15^ FEV_1_/FVC ratio greater than 0.7. The exam was always conducted by the same evaluator and the data obtained were part of the sample characterization. Children who did not meet the inclusion criteria were not selected for the study.

Exclusion criteria were the inability to adequately perform any of the proposed procedures, altered IOS parameters^9^ and presence of acute respiratory disease on the day of data collection. All parents/guardians signed the informed consent form on behalf of participants.

**Figure 1:**
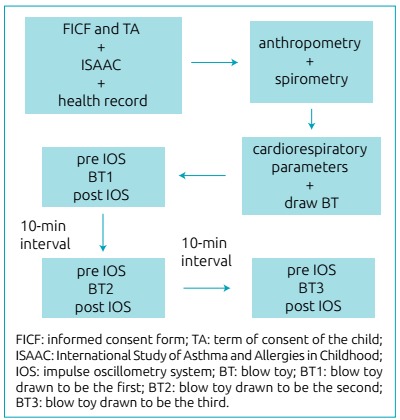
Flowchart of the data collection procedures.

The organization chart of procedures performed is shown in Figure 1. Initially, the participants filled out a standardized evaluation form and were submitted to anthropometric mass evaluation (in kilograms) using a previously calibrated digital scale (ISP^®^ brand, São Paulo, Brazil), with capacity of 180 kg and precision of 100 g, and height measurement (in centimeters, cm), by a fixed stadiometer (Filizola^®^, São Paulo, Brazil). These measurements were collected in an isolated room, with the participant in upright and aligned posture, barefoot and wearing light clothing. After obtaining height and weight data, the body mass index (BMI) was calculated in kg/m^2^, according to the National Telehealth Brazil Networks Program.^16^

For randomization, a simple draw was made to determine the order of use of each blow toy. Each child picked one from three sealed envelopes, each named after one of the three toys in succession. The child chose one envelope at a time this determined the order of their use. The participants were then explained about the respiratory system tests (IOS and spirometry) and the three blow toys: party whistle (Festalita, São Paulo, Brazil), soap bubbles (Magic bublle, Brasilflex^®^, São Paulo, Brazil) and balloon (Ballontech^®^, São Bernardo do Campo, Brazil). For this, the evaluator explained and demonstrated the procedures and respiratory maneuvers involved. The three toys were in compliance with the specifications of the National Institute of Metrology, Standardization and Industrial Quality - INMETRO (INMETRO Ordinance No. 006/2011).

The use of each blow toy was as follows:

- Soap bubbles: the child was instructed to perform normal breathing with tidal volume (TD) inspiration and slow mouth exhalation for at least six seconds and with laminar flow. Verbal command was given for them to make “big bubbles”. Each child performed ten consecutive breathing cycles, whether or not soap bubbles were formed.
- Party whistle: a medium volume inhalation was requested, followed by an average force and velocity mouth exhalation for at least three seconds to beat the R of the toy and unfold it completely. Each child performed ten consecutive breathing cycles, whether or not they succeeded in unfolding the toy.
- Balloon: a deep nasal inspiration, close to total lung capacity (TLC), was requested, followed by an oral exhalation with enough force and speed to overcome the R of the toy. Each child performed ten consecutive breathing cycles, regardless of whether or not the toy was filled (partially or completely).

Before and immediately after the use of each toy, the cardiorespiratory parameters of heart rate (HR), respiratory rate (RR) and O_2_ saturation (SpO_2_) were recorded and the evaluation of respiratory mechanics was conducted by IOS (Master Screen IOS, Eric Jaeger, Germany^®^) (Figure 1), according to the American Thoracic Society’s indications,^17^ always by the same researcher. Three IOS measurements were performed, with records of 30 seconds duration and 30 seconds interval between each. Based on graphic records, those who had no cough, swallowing or speaking during the maneuver were admitted. The first valid record^14^ ^,^ ^18^ ^,^ ^19^ was analyzed as long as the three maneuvers had been reproducible, that is, the values did not vary by more than 10% between them.

The oscillometric variables analyzed were Z, resistance at 5 hertz (R5 **-** compatible with total airway resistance), resistance at 20 hertz (R20 **-** central airway resistance), respiratory reactance at 5 hertz (X5) and AX, expressed as absolute values and percentages of predicted values, according to Assumpção et al.^9^

For sample calculation, the G*Power software was used. The R5 parameter was chosen for analysis, with a small effect and partial Eta-squared of 0.1. To achieve a test power of 80% and significance level of 5%, the calculation pointed to a sample of 46 students. Adding to this calculation a sample buffer of 10%, 51 individuals in each group were considered enough for the research.

All data were input to a Microsoft Excel^®^ spreadsheet and transported to a database in IBM SPSS™ 20.0 (New York, United States) for Windows^®^ for further analysis.

Descriptive and frequency statistics were calculated, with data presented as mean, standard deviation, median, minimum and maximum. Initially, the Kolmogorov-Smirnov test was applied and, depending on the result, the analysis of variance (ANOVA) was applied for repeated measures to compare the moments before and between uses of blow toys, with location of differences calculated by Bonferroni test and post-hoc. The results of the ANOVA test for repeated measures were presented as mean, standard deviation, degree of freedom and effect size. Significance level was set at 5% (p≤0.05) in all tests.

The procedures were always performed by the same researchers, who had been previously trained, completed an evaluation form, and conducted anthropometry.

## RESULTS

We evaluated 105 children, 34 of which were not compatible with the inclusion criteria (abnormal spirometry, altered ISAAC). Thus, 71 healthy students took part in the sample, most of them being females (40). Of the total participants, 47 were normal weight, one was underweight, 17 were overweight and six were obese (Table 1). No child was excluded from the survey.

**Table 1:** Characterization of the sample as to age, weight, height and body mass index.

	Mean	SD	Median	Min.	**Max.**
Age (years)	9.7	2.1	9.0	7.0	14.0
Weight (kg)	37.6	10.1	36.2	20.0	66.7
Height (cm)	141.8	11.9	141.0	116.0	163.0
BMI (kg/m^2^)	18.1	3.11	17.4	12.8	26.4

SD: standard deviation; BMI: body mass index; kg: kilogram; cm: centimeter; m^2^: square meter.

There was a statistically significant difference in the gross values of the oscillometric parameters of R5, Fres and AX in the moment before, between the first and third blow toys (R5/p=0.050; Fres/p=0.008; AX/p=0.016). In the Fres parameter, the difference also happened between the first and the second toy (p=0.041). When comparing the predicted percentage values of each oscillometric parameter before the use of each toy, there was a difference in respiratory impedance parameters at 5 hertz (Z5), R5 and Fres in the moment before between the first and the third toy. In Z5, there was a difference in the moment before between the first and third wind toy (Z5/p=0.048; R5/p=0.049; Fres/p=0.004), and in the Fres parameter, the moment before between the first and the second toy (p = 0.048) also differed (Table 2).

**Table 2:** Comparison of the gross values and % of predicted oscillometric parameters among the moments before the use of the three blow toys.

		Mean±SD	F	DF	Partial Eta	p-value
Z5	BT1	0.6±0.1				
	BT2	0.6±0.1	3.9	1.7; 124.4	0.053	0.062
	BT3	0.6±0.1				
Z5 predicted %	BT1	155.4±37.8				
	BT2	160.7±42.3	3.1	2; 140	0.044	0.044*
	BT3	147.9±37.2				
R5	BT1	0.6±0.1				
	BT2	0.6±0.1	4.2	1.7; 121.8	0.057	0.022*
	BT3	0.5±0.1				
R5 predicted %	BT1	106.2±20.3				
	BT2	103.1±20.7	3.9	1.7; 123.0	0.054	0.026*
	BT3	101.1±19.8				
R20	BT1	0.4±0.1				
	BT2	0.4±0.9	0.8	2; 140	0.012	0.428
	BT3	0.4±0.8				
R20 predicted %	BT1	101.3±18.9				
	BT2	99.4±18.7	0.8	2; 140	0.012	0.431
	BT3	99.7±18.1				
X5	BT1	-0.1±0.7				
	BT2	-0.2±0.1	0.7	2; 140	0.011	0.460
	BT3	-0.2±0.07				
X5 predicted %	BT1	124.4±41.2				
	BT2	127.4±42.9	0.8	2; 140	0.012	0.428
	BT3	121.9±38.9				
Fres	BT1	18.3±5.4				
	BT2	17.4±5.0	6.4	2; 140	0.084	0.002*
	BT3	17.0±5.2				
Fres predicted %	BT1	111.7±29.1				
	BT2	106.5±27.3	7.1	2; 140	0.092	0.001*
	BT3	103.6+26.3				
AX	BT1	1.3±1.0				
	BT2	1.1±0.9	5.7	1.7; 121.1	0.076	0.006*
	BT3	1.1±0.9				
AX predicted %	BT1	132.2±103.4				
	BT2	132.0±117.7	0.3	1.3; 92.6	0.005	0.606
	BT3	118.9±93.0				

Z5: respiratory impedance at 5 Hz (kPas/L/s); R5: resistance at 5 Hz (kPas/L/s); R20: resistance at 20 hertz (kPas/L/s); X5: reactance at 5 hertz (kPas/L/s); Fres: resonant frequency (1/s); AX: reactance area (kpa/L); SD: standard deviation; F: ratio F; Df: degree of freedom; partial Eta: effect size; p-value: significance level according to the repeated measures ANOVA test (*p<0.05) and Bonferroni post-hoc test.

The oscillometric parameters, in gross value and percentage predicted, listed in Table 3, when compared between right after the use of each toy were not statistically different (Table 3).

**Table 3:** Comparison of the gross values and % of predicted oscillometric parameters among the moments after the use of the three blow toys.

		Mean±SD	F	DF	Partial Eta	p-value
Z5	BT1,1	0.6±1.8				
	BT2,1	0.6±0.1	0.38	2; 140	0.005	0.684
	BT3,1	0.6±0.1				
Z5 predicted %	BT1,1	161.9+44.9				
	BT2,1	160.7+43.3	0.1	2; 140	0.002	0.889
	BT3,1	161.4±42.0				
R5	BT1,1	0.6±0.1				
	BT2,1	0.6±0.1	0.1	2; 140	0.003	0.826
	BT3,1	0.6±0.1				
R5 predicted %	BT1,1	110.0±24.2				
	BT2,1	109.3±21.3	0.1	2; 140	0.001	0.911
	BT3,1	109.4±21.9				
R20	BT1,1	0.5±0.1				
	BT2,1	0.5±0.1	0.02	1.7; 123.5	0.001	0.979
	BT3,1	0.5±0.9				
R20 predicted %	BT1,1	102.5±20.8				
	BT2,1	102.8±20.5	0.02	1.8; 169.3	0.001	0.964
	BT3,1	102.3±21.6				
X5	BT1,1	-0.1±0.1				
	BT2,1	-0.1±0.8	0.4	2; 140	0.006	0.635
	BT3,1	-0.1±0.8				
X5 predicted %	BT1,1	130.8±60.6				
	BT2,1	125.5±50.3	0.4	2; 140	0.006	0.626
	BT3,1	126.6±53.8				
Fres	BT1,1	18.7±5.4				
	BT2,1	18.5±5.7	0.4	2; 140	0.007	0.631
	BT3,1	18.4±5.4				
Fres predicted %	BT1,1	113.5±28.0				
	BT2,1	113.0±28.9	0.1	2; 140	0.002	0.871
	BT3,1	112.2±28.5				
AX	BT1,1	1.4±1.1				
	BT2,1	1.3±1.2	1.7	2; 140	0.024	0.182
	B3,1	1.3±1.0				
AX predicted %	BT1,1	157.3±141.0				
	BT2,1	147.0±121.3	1.2	2; 140	0.018	0.286
	BT3,1	120.3±129.3				

Z5: respiratory impedance at 5 Hz (kPas/L/s); R5: resistance at 5 Hz (kPas/L/s); R20: resistance at 20 hertz (kPas/L/s); X5: reactance at 5 hertz (kPas/L/s); Fres: resonant frequency (1/s); AX: reactance area (kpa/L); SD: standard deviation; F: ratio F; Df: degree of freedom; partial Eta: effect size; p-value: significance level according to the repeated measures ANOVA test (*p<0.05) and Bonferroni post-hoc test.

## DISCUSSION

Blow toys are present in clinical practice and their use in respiratory physiotherapy makes it possible to teach and perform breathing exercises in a playful and enjoyable manner.^20^ However, one must understand whether the order of execution or the sum of ventilatory stimuli during physical therapy alters respiratory mechanics parameters, and this prompted the current investigation. The present study stands out for its unprecedented investigations of this repercussion and showed that the overlapping of stimuli with by using blow toys provided an improvement in the airway R of healthy children, with reduction, in the moment before, between the first and third toys, in Z5, R5 and Fres.

The Z5 parameter represents all mechanical load by the respiratory system and consists in the sum of the parameters of R and X.^21^ In the present investigation, there was a reduction in Z5, probably as a consequence of the reduction in R5. This represents the total airway R,^22^ and the fact that there was no change in R20 related to the central airway R indicates that blow toys have a positive effect on the most peripheral portion of the bronchial tree. This information can be reinforced by the Fres parameter, which refers to peripheral airway behavior and has also shown improved values after intervention.^22^

The progressive reduction of Z5, R5 and Fres parameters suggests that blow toys are capable of reducing airway R; therefore, a possible phenomenon of pulmonary deflation should also be investigated in the future. The other parameters and the analysis of the moments after the use of the three blow toys did not vary. This may have stemmed from the maintenance of the effects of toys after use, which is identified in the analysis of the moment before, or because the use of these toys was fast and included few repetitions, which can be considered a limitation of study, as well as the fact that the sample was composed of individuals without respiratory impairments. Also regarding the sample, it is relevant to justify the presence of obese children, since altered IOS parameters were identified in this population.^23^ Children with this profile were kept in the sample because they were compatible with the inclusion criteria and values within the normal range, according to reference values for Brazilian children.^9^ It should be noted that this study is characterized by having a convenience sample, in which two children (11 and 13 years old), upon performing spirometry, showed altered values ​​and were excluded. This may be justified by the difficulty in performing the exam, which requires the great collaboration of the individual,^24^ and not necessarily the presence of a respiratory disease.

IOS evaluates respiratory mechanics noninvasively and has been applied as a diagnostic tool, which provides assessment of pulmonary function, specifically respiratory mechanics, of individuals with asthma, cystic fibrosis, and other respiratory disorders.^24^ ^,^ ^25^ ^,^ ^26^ To date, the literature has no studies investigating the effects of respiratory physiotherapy techniques on respiratory mechanics. Breathing exercises performed through nasal inspiration and slow and prolonged exhalation, such as the lip frenum, are expected to increase VT, promote pulmonary deflation and airway stability.^27^ ^,^ ^28^ These effects, however, have not yet been fully elucidated in isolation or in association with wind toys.

Considering the purpose of each blow toy and the execution according to previous guidelines, it is suggested that physical therapy sessions involving the use of blow toys promote therapeutic protocols that stimulate different lung flows and volumes with assorted tools alike. Importantly, the three blow toys used in this study, as well as most secretion removal techniques, stimulate the expiratory phase, which is of great importance in physical therapy care.

In this research, the use of blow toys also motivated children, from the youngest to the oldest, to perform specific breathing patterns. Participants identified the successful execution of toys by forming soap bubbles, stretching the party whistle’s filament and inflating balloons, which was attributed to the prior systematized guidance they received for use. This behavior equals greater involvement of participants, which can have a positive effect on respiratory physiotherapy. In addition, the selected toys were simple and inexpensive features, including the whistle and the pinwheel, besides being familiar and applicable to the therapeutic routine, even with home extension, compared to more expensive and more complex devices.

Costa et al.^8^ have already found how beneficial recreational resources are and their relevance when it comes to pediatric physical therapy rehabilitation. These authors developed a protocol for the use of blow toys for maneuvers and breathing techniques. The toys used were straw, party whistle and soap bubbles, in order to allow the performance of breathing exercises. The results showed that the children in the group undergoing physical therapy with toys were more collaborative during therapy and showed reduced stress. These results corroborate what has been observed in clinical practice and discussed in the literature.^29^

Finally, it was found that the overlap of different blow toys seems to have potential effect on the respiratory mechanics of healthy children, since the sum of ventilatory stimuli reduced airway resistance, identified by the difference in Fres and R5 parameters before the use of each toy. Little is known about the use of toys, games and play in the clinical practice of the physical therapist, which adds great relevance to the present study, providing knowledge and scientific support for the use of these toys in clinical practice. From this work, others may emerge aiming to verify physiological and ventilatory responses in children with respiratory impairment.
